# Long-Term Impedance Trends Over 10 Years Following Cochlear Implantation: A Retrospective Comparative Analysis

**DOI:** 10.1177/19160216261442102

**Published:** 2026-09-28

**Authors:** Jianhang Deng, Martin Koch, Matthias Bornitz, Sijia Zhai, Marcus Neudert, Till Moritz Essinger

**Affiliations:** 1 Ear Research Center Dresden, Department of Otorhinolaryngology, Head and Neck Surgery, Faculty of Medicine and University Hospital Carl Gustav Carus, TUD Dresden University of Technology, Dresden, Germany

**Keywords:** cochlear implant (CI), electrode impedance, longitudinal analysis, pediatric implantation, electrode design

## Abstract

**Importance:**

Impedance monitoring is a critical component of cochlear implant (CI) surgery, yet the lack of comprehensive longitudinal data limits the development of evidence-based postoperative monitoring guidelines, potentially compromising patient outcomes.

**Objective:**

To track impedance trends over 10 years post-implantation, evaluate the effects of electrode design on impedance dynamics, and examine the influence of patient age on impedance behavior.

**Design:**

Retrospective cohort study.

**Setting:**

Tertiary medical center.

**Participants:**

A total of 554 ears underwent cochlear implantation between June 2006 and December 2014; each ear received 1 electrode array from 1 of 6 electrode types.

**Exposure or Intervention:**

Longitudinal impedance data were analyzed across apical, medial, and basal electrode contacts at 7 time points.

**Main Outcome Measures:**

Impedance trajectories varied by age group, electrode design, and cochlear region, with persistent changes observed up to 10 years.

**Results:**

Impedance increased markedly from intraoperative to first activation in most arrays, although a significant decrease in apical contacts was observed in CI512 adults (*P* = .0008). Apical and medial contacts generally stabilized or declined thereafter. In contrast, basal electrodes exhibited more heterogeneous patterns, including increases during 12 to 60 months of follow-up and decreases during 60 to 120 months. Compared to PM arrays, LW electrodes showed higher apical impedances, medial impedances that were mostly comparable and time-dependent, and persistently lower basal impedances than CI512. Among LW arrays, CI422 consistently demonstrated the highest impedance, whereas the MED-EL electrode series, Flexsoft exhibited the highest impedance. Overall impedances were higher in early childhood recipients, while adults tended to show higher values in the basal region.

**Conclusion:**

This study provides the first decade-long characterization of impedance changes, revealing dynamic variations in electrode impedance across different designs and age groups of cochlear implant recipients.

**Relevance:**

This study highlights the need for long-term monitoring and offering practical guidance for individualized programming and electrode design.

## Graphical abstract

**Graphical Abstract:**
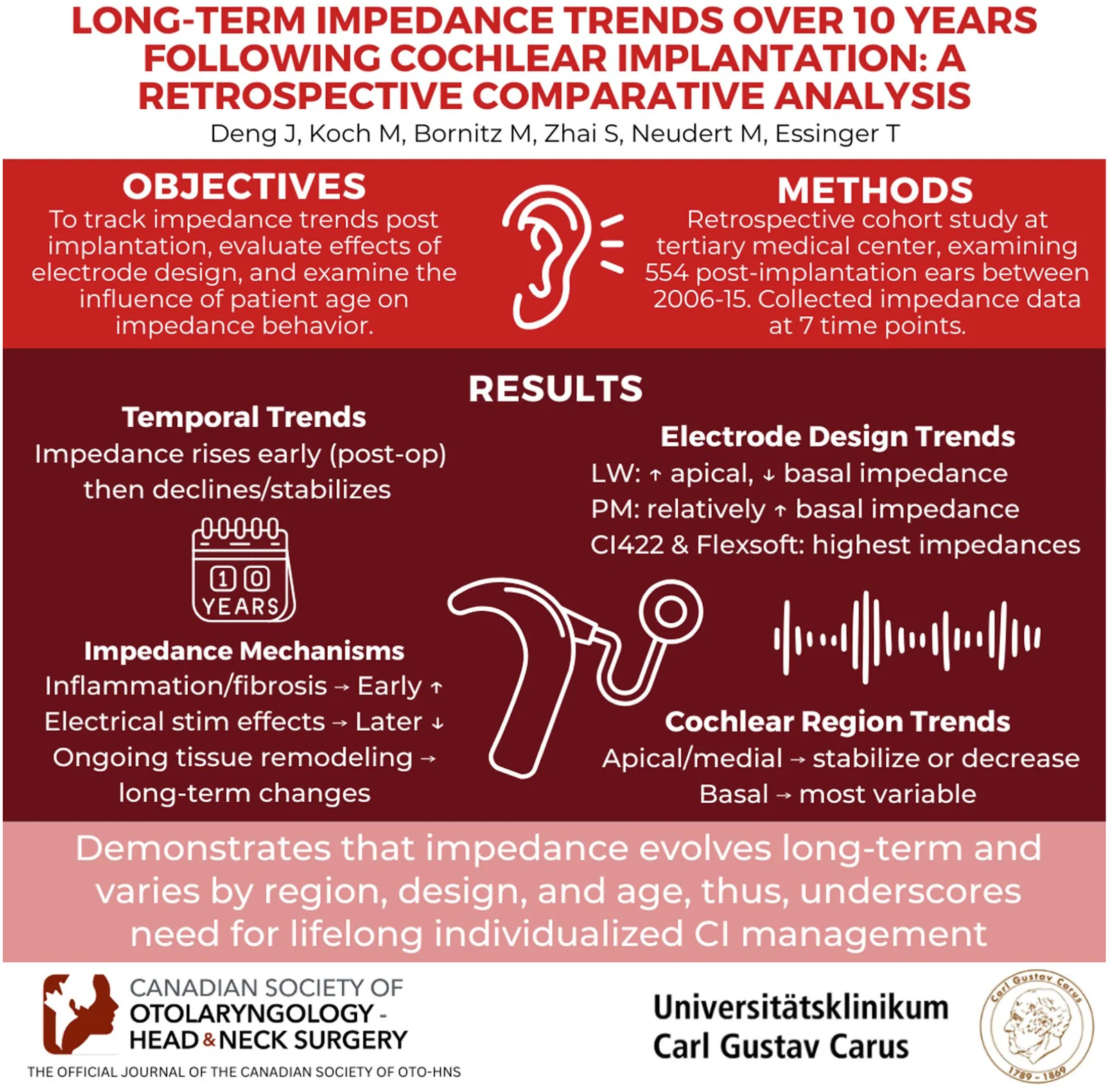

## Key message

- Provides a large-cohort, decade-long characterization of region-specific impedance trajectories in cochlear implant recipients.
- Demonstrates that the effects of electrode design and patient age on impedance are time- and region-dependent, underscoring the need for individualized, longitudinal management strategies.

## Introduction

Cochlear implants (CI) represent the most successful sensory neuroprosthetic device to date, benefiting approximately 800,000 recipients worldwide.^1^ Device programming relies heavily on objective measures to verify internal function and optimize auditory access. Among these measures, electrode impedance serves as a universal and rapid indicator of electrode–tissue interface status across all manufacturers, despite differences in terminology or implementation.^2,3^

Impedance reflects near-field and far-field components,^4,5^ and is essential both intraoperatively and postoperatively, where it enables confirmation of electrode position and early detection of tissue responses such as fibrosis, inflammation, edema, scarring, and ossification.^6-9^ Because impedance influences stimulation levels, power consumption, and ultimately auditory outcomes,^10-14^ understanding its long-term behavior is critical for guiding CI management.

However, existing evidence is dominated by short-term postoperative data. Only 3 studies have extended beyond 8 to 10 years, and these were limited by sparse sampling or severe attrition. Newbold et al^15^ reported only 2 discrete time points, missing the continuous temporal evolution of impedance. Konrad et al^16,17^ examined Hybrid-L24 electrodes longitudinally, but only 2 participants remained at the 10-year follow-up, precluding meaningful statistical conclusions. As a result, robust long-term trajectories of CI impedance remain largely uncharacterized, restricting our ability to establish evidence-based monitoring guidelines.

Emerging findings suggest that factors such as electrode design and patient age may critically shape impedance evolution,^18-20^ yet the mechanisms underlying these variations are poorly understood.

This study aims to address these gaps by (1) longitudinally tracking impedance trends over a 10-year period following implantation, (2) analyzing the effects of different electrode designs on impedance dynamics, and (3) exploring the influence of patient age on impedance behavior.

## Materials and Methods

This study was approved by the ethics committee at TU Dresden, reference number BO ff (Mono)-EK-357082025.

### Participants

Data of cochlear implantation procedures performed from June 2006 to December 2014 were comprehensively reviewed. Eligibility was assessed at the ear level. We first screened ears implanted with 1 of the prespecified electrode arrays with available impedance record (MED-EL Flex28/Flexsoft/Standard or Cochlear Nucleus CI422/CI24RE (CA)/CI512; Figure 1).

**Figure 1.**
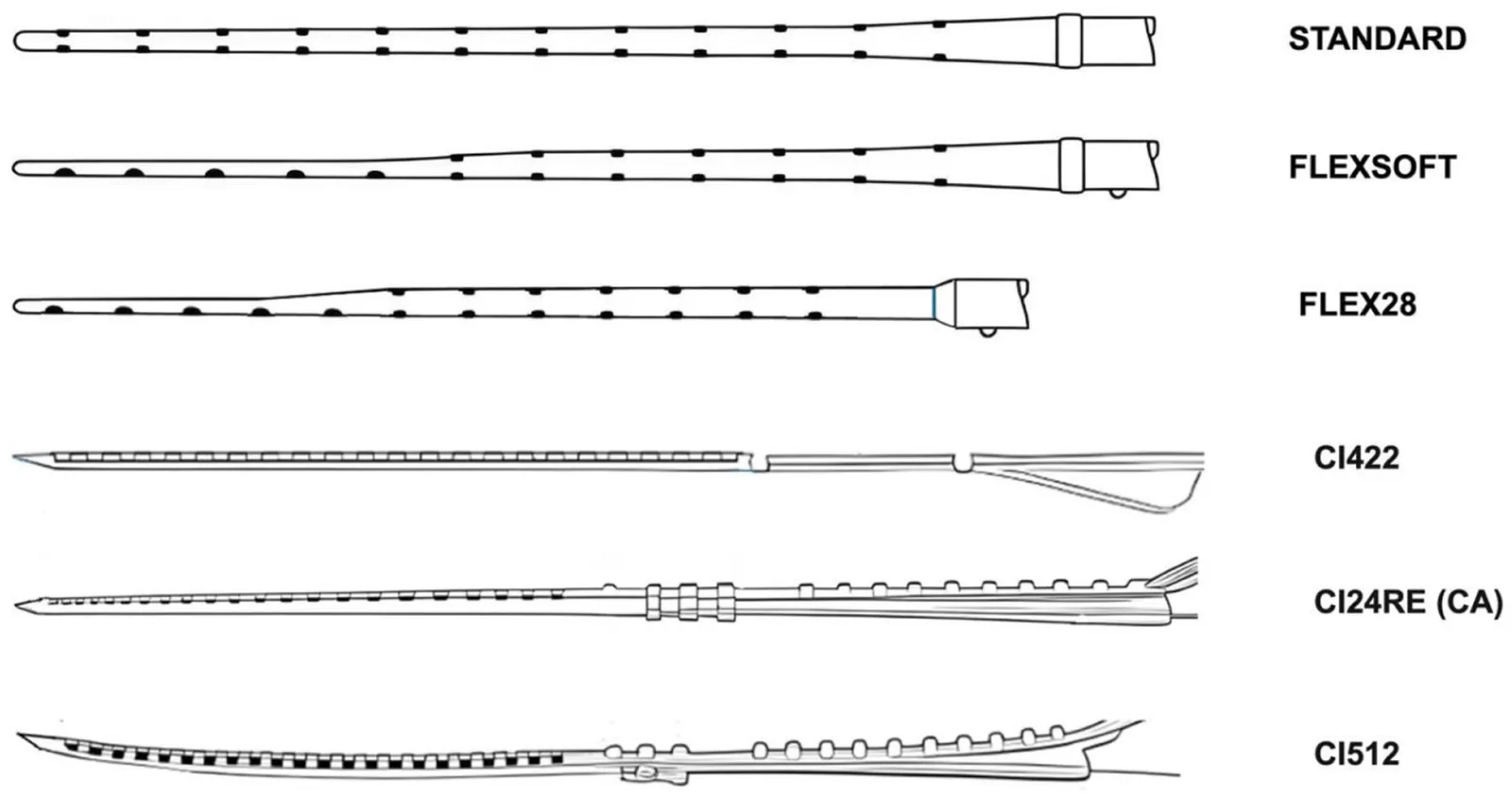
Schematic drawing of electrode array types.

Exclusion criteria (ear-level) were applied sequentially as follows: (1) implantation age 7 to 17 years; (2) any ear with ≥1 electrode contact deactivated (“off/disabled”) at any time during follow-up, as documented in the manufacturer software and saved programming (MAP) records.

### Surgical Procedure and Management

All patients underwent standard preoperative evaluations. Surgeries were performed by experienced otologic surgeons following standardized protocols with minimally invasive approach. Postoperative management adhered to established clinical guidelines, including implant activation at 1-month post-implantation, hearing rehabilitation and regular long term follow-ups to ensure consistent care and optimal outcomes. Postoperative imaging was routinely performed on postoperative day 1, preferentially using cone-beam CT (digital volume tomography [DVT]). If DVT was not feasible, a plain radiograph in the Altschul-Uffenorde projection was obtained.

### Impedance Measurements

Impedance field telemetry in MED-EL recipients was acquired using the manufacturer’s standardized clinical protocol implemented in the MAESTRO software suite (MED-EL Medical Electronics). For Cochlear Nucleus participants, impedance measurements were obtained through Custom Sound (Cochlear Ltd.). Monopolar 1 + 2 (MP 1 + 2) electrode configurations were analyzed.

Intraoperative and longitudinal postoperative data were extracted from device programming logs. Impedance values were analyzed at the following postoperative intervals (months): 0 (intraoperative), 1 (activation), 6 ± 1, 12 ± 2, 24 ± 4, 60 ± 10, and 120 ± 20. At the activation visit, impedance was defined as the first impedance telemetry recorded prior to electrical stimulation during the initial activation/mapping session in the manufacturer’s fitting software. To accommodate routine variability in clinical follow-up, measurements obtained within the predefined deviation window were accepted as valid representations of the target time point. When no measurement was available within this window, the corresponding time point for that ear was omitted from the analysis. Intraoperative impedance recordings showing electrode isolation consistent with air-bubble artifacts were excluded for the intraoperative time point only; postoperative measurements for the same ear were retained.

### Statistical Analysis

Computed mean impedance (kΩ) for apical/medial/basal segments was modeled using linear mixed-effects models in R (lme4).^21^

Time was modeled with a natural cubic spline (knots: 1, 6, 60 months). Random effects were a patient intercept and time slope, plus an ear-within-patient intercept. Fixed effects included hardware (tested separately due to non-independence: electrode type, manufacturer, and array design [lateral wall vs perimodiolar]), segment, age group, and interactions up to three-way with time. Model selection used Akaike information criterion^22^ and patient-wise 5-fold cross-validation (Root mean square error ± standard deviation [SD]). We report marginal/conditional R²,^23^ variance components, and Estimated marginal means (EMMs) with Tukey-adjusted contrasts^24^ ( Supplemental Appendix S1).

## Results

### Demographics

Table 1 shows study inclusion/exclusion and follow-up availability. This study included a total of 554 cochlear implant ears (see Table 2), focusing 6 different electrode types from 2 manufacturers. The electrodes from MED-EL were all lateral wall (LW) arrays, each featuring 12 electrode contacts (S1-S12), while the Cochlear Nucleus electrodes, with 22 electrode contacts (E22-E1), included both LW (CI422) and perimodiolar (PM) designs (CI512 and CI24RE (CA)). All participants were categorized into adult and early childhood groups for analysis. Implanted age is presented as mean ± SD in years. Follow-up availability by device and age group is summarized in Supplemental Table S1.

**Table 1.** Selection and Exclusion of Study Ears.

Step	Exclusion category	Excluded, N	Remaining, N
1	Ears screened: study electrode arrays with available impedance record (Jun 2006-Dec 2014)	/	709
2	Excluded: ineligible age at implantation (7-17 years)	39	670
3	Excluded: ≥1 electrode contact deactivated (“off/disabled”) at any time during follow-up (based on saved MAP/programming records)	116	554

*N* denotes the number of ears.

**Table 2.** Demographic information on participant ears.

Manufacturer	MED-EL			Cochlear nucleus		
Adult participants						
Subject group	Flex28	Flexsoft	Standard	CI422	CI512	CI24RE(CA)
Design type	Lateral wall	Lateral wall	Lateral wall	Lateral wall	Perimodiolar	Perimodiolar
Electrode shape	Straight	Straight	Straight	Straight	Pre-curved	Pre-curved
Active length (mm)	23.1	26.4	26.4	20	15	15
Electrode sites	12	12	12	22	22	22
Number of ears	54	96	79	114	36	46
Implanted age (years)	61.48 ± 15.64	61.55 ± 13.71	55.04 ± 16.48	56.70 ± 15.46	49.14 ± 17.73	56.63 ± 11.88
Male/female	23/31	47/49	27/52	58/56	23/13	18/28
Right/left	27/27	48/48	46/33	55/59	18/18	21/25
Early childhood participants						
Subject group	Flex28	Flexsoft	Standard	CI422	CI512	CI24RE(CA)
Design type	Lateral wall	Lateral wall	Lateral wall	Lateral wall	Perimodiolar	Perimodiolar
Electrode shape	Straight	Straight	Straight	Straight	Pre-curved	Pre-curved
Active length (mm)	23.1	26.4	26.4	20	15	15
Electrode sites	12	12	12	22	22	22
Number of ears	15	13	28	27	22	24
Implanted age (years)	0.73 ± 0.79	1.31 ± 0.48	2.04 ± 1.88	1.59 ± 1.76	1.82 ± 1.44	1.75 ± 1.54
Male/female	9/6	9/4	16/12	15/12	9/13	17/7
Right/left	8/7	7/6	15/13	12/15	11/11	14/10

### Model Validation and Summary of the Mixed-Effects Analysis

The final linear mixed-effects model retained exact follow-up time (days) and modeled time flexibly as a natural cubic spline f(t) on a log-transformed time scale (Supplemental Appendix S1). Fixed effects included [f(t) × Type × Place], [Age × f(t) × Place] and [Age × Type], with random intercept and time slope and an ear-within-patient intercept. Model performance was marginal R² = 0.58 and conditional R² = 0.84. Variance components were 11.6% (between-patient intercept), 27.6% (ear-within-patient), 5.9% (between-patient slope), and 55.0% (residual), indicating that ear-level variability exceeded baseline between-patient variability. EMMs and Tukey-adjusted contrasts are reported in Supplemental Appendix S1.

### Longitudinal Impedance Dynamics: From Intraoperative to 120 Months Post-Implantation

#### General Temporal Pattern

In addition to the statistically significant changes described (Impedance variations across different time intervals), several broader temporal and distributional trends can be observed across electrode designs and patient groups (Figure 2). LW electrodes showed a more compact impedance distribution across timepoints compared to PM arrays. In the early postoperative period, LW electrodes consistently displayed higher impedance in the apical region, whereas PM arrays tended to show higher impedance in the basal region, with the exception of the intraoperative measurement. Within the LW group, early childhood recipients consistently exhibited higher impedance in apical and medial electrodes compared to adults, resulting in a broader impedance distribution along the array; However, this discrepancy diminished over time, with values converging toward the 120-month follow-up. Notably, apical electrodes remained the highest-impedance region throughout follow-up across all early childhood LW cohorts.

**Figure 2.**
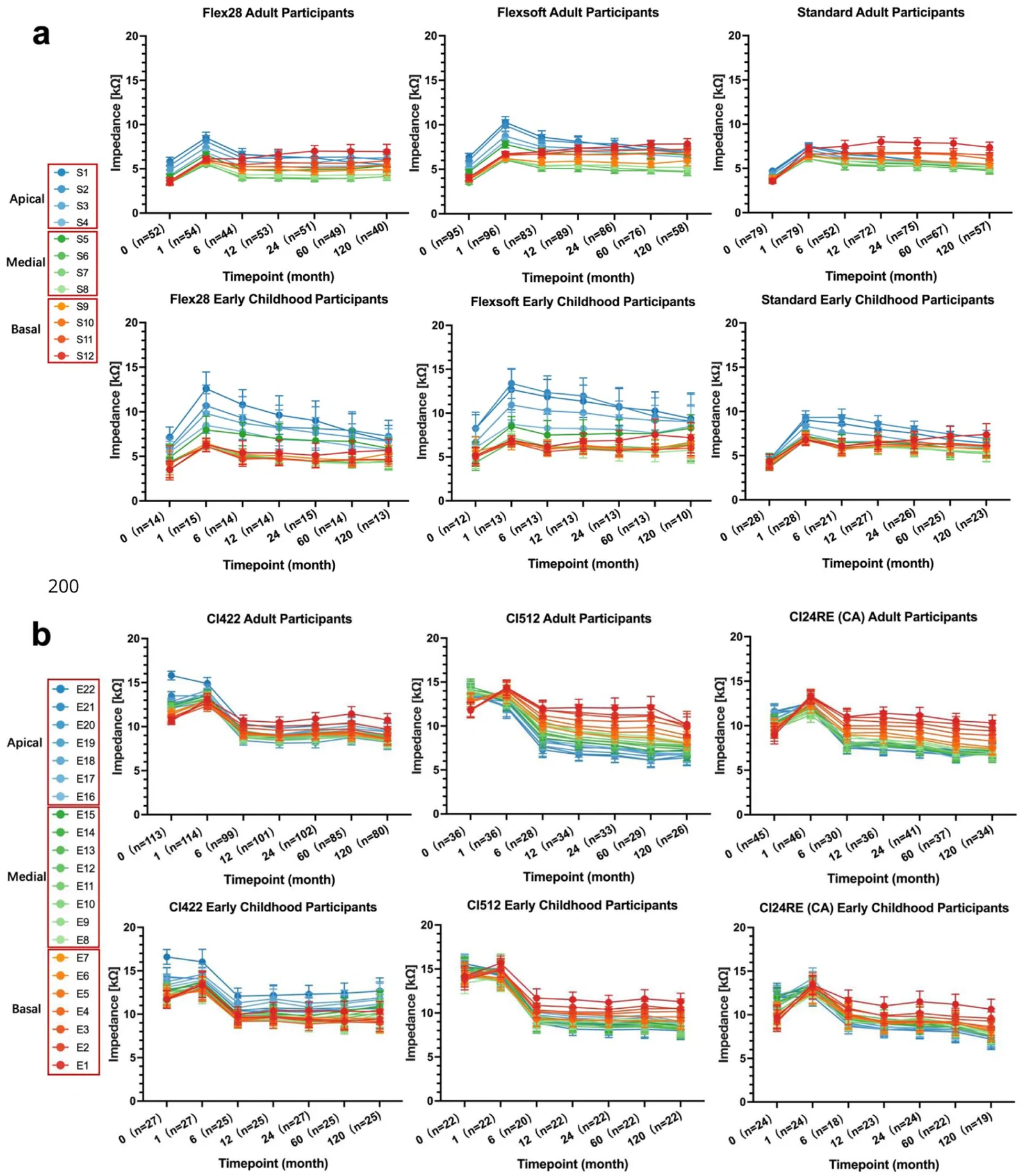
Electrode impedance evolution over 120 months post-implantation in adult and early childhood participants with (a) MED-EL (Flex28, Flexsoft, Standard) and (b) Cochlear Nucleus (CI422, CI512, CI24RE (CA)) electrodes. Timepoints (0, 1, 6, 12, 24, 60, 120 months) represent follow-up months. The x-axis sample size (n) indicates the number of ears at each timepoint. Electrode section: MED-EL: S1 to S4 (apical), S5 to S8 (medial), S9 to S12 (basal). Cochlear Nucleus: E22 to E16 (apical), E15 to E8 (medial), E7 to E1 (basal). Data are presented as mean ± 95% confidence interval.

#### Impedance Variations Across Different Time Intervals

The impedance values of the apical, medial, and basal sections of all MED-EL electrodes increased significantly from the intraoperative period to 1 month postoperatively. Thereafter, apical and medial impedances generally declined during the first postoperative year and were generally stable thereafter, with array- and age-specific changes during mid-term follow-up. Notably, between 60 and 120 months post-implantation, apical impedance decreased further in Flex28 adults (*P* = .0238) and in the Flexsoft cohort (adults: *P* = .0002; early childhood: *P* = .0060), while medial impedance showed an additional decline in Flexsoft adults (*P* = .0289). In contrast, basal impedance demonstrated more heterogeneous early trajectories and was generally stable at later intervals, with selected exceptions; a significant late decrease from 60 to 120 months was observed in Standard adults (*P* = .0246).

For Cochlear Nucleus electrodes, apical impedance decreased in CI512 adults from the intraoperative measurement to 1 month postoperatively (*P* = .0008), whereas apical impedance increased in all CI422 and CI24RE(CA) participants. In the medial segment, impedance remained stable in CI512 but increased significantly in CI422 and CI24RE(CA). Basal impedance increased significantly in all cohorts. Following this initial phase, impedances generally declined across cochlear segments through 12 months, followed by more heterogeneous mid- to long-term patterns, including significant increases in CI422 between 12 and 60 months and selective late decreases by 120 months in several segments. Detailed demographic data are provided in Supplemental Table S2.

#### Electrode Array Type-Related Impedance Differences

##### Comparison of PM and LW Electrode Arrays

In Cochlear Nucleus electrodes, the impedance values of apical, medial, and basal electrodes were compared between PM and LW arrays across different time points (Figure 3). Apically, LW demonstrated higher overall impedances than the PM array. Across all participants, impedance values for CI422 were higher than those for CI24RE(CA) at all time points, with the only non-significant difference observed in the early childhood group at 6 months postoperatively. Relative to CI512, apical impedances were nonsignificant intraoperatively and at 1 month in adults but were higher for CI422 from 6 months onward; in the early childhood group, differences were nonsignificant through 12 months and became higher for CI422 from 24 months onward. Medially, CI422 was generally higher than CI24RE (CA), reaching significance only at selected intervals. In contrast, CI422 to CI512 differences were time dependent: CI422 was lower intraoperatively in both groups, largely nonsignificant postoperatively, and higher only at late follow-up in adults (60, 120 months). Basally, CI422 remained lower than CI512 across all time points, with attenuation over time and predominantly nonsignificant differences by 60 to 120 months. Compared with CI24RE (CA), CI422 was significantly higher intraoperatively, whereas postoperatively CI24RE(CA) tended to be higher, becoming significant at 6 months in adults. Detailed demographic data are provided in Supplemental Table S3.

**Figure 3.**
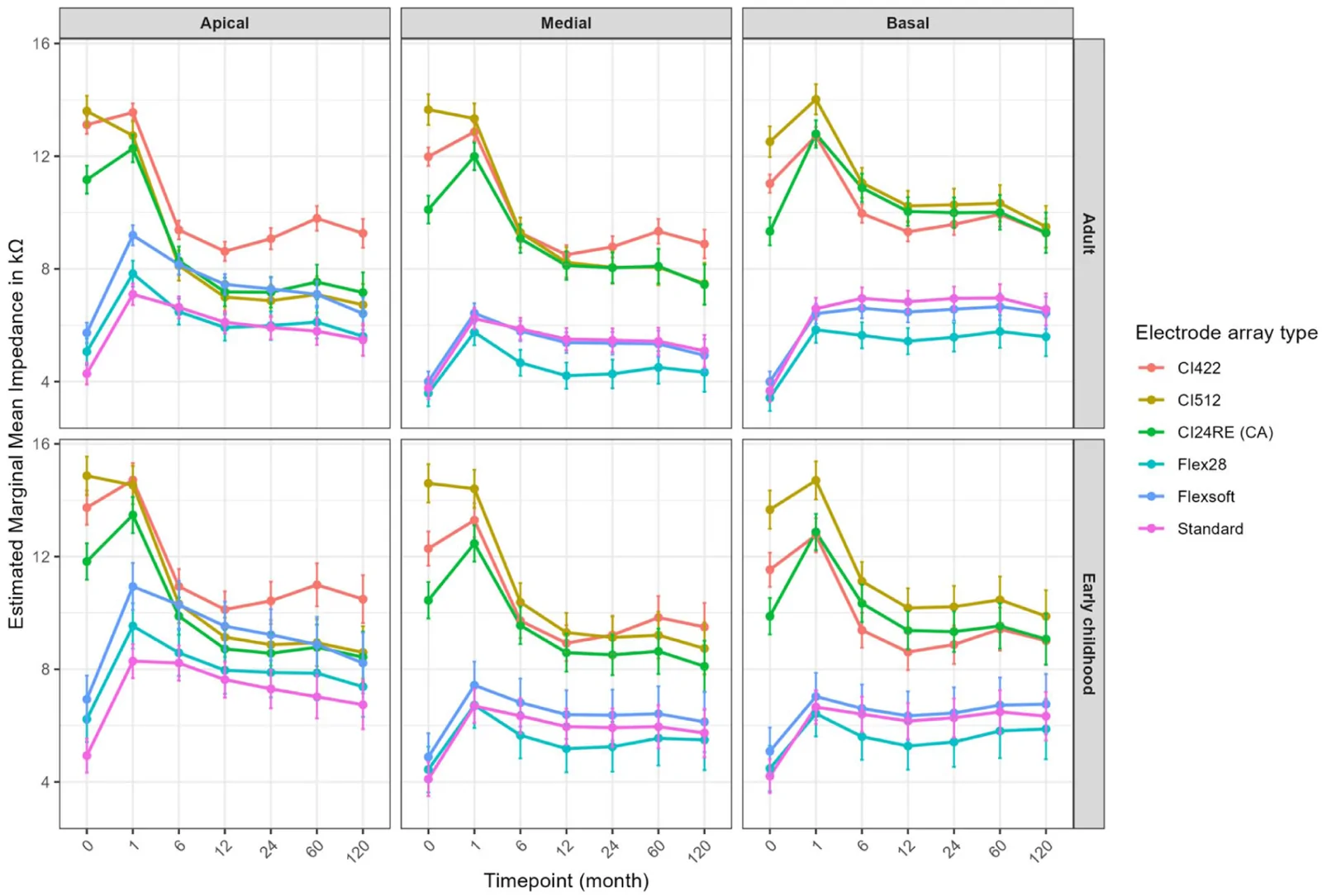
Estimated Marginal Mean impedance values (kΩ) of each electrode array type by age groups across different time points and electrode sections. Data are presented as mean ± 95% confidence interval.

##### Comparison Among LW Electrode Arrays

We compared the impedance values of LW electrodes from 2 manufacturers. Across all time points and electrode sections, both adult and early childhood participants consistently exhibited significantly higher impedance in CI422 electrodes compared to Flexsoft, Flex28, and Standard arrays (Figure 3).

Within the MED-EL electrode series, segment-specific differences were driven primarily by the apical region. In both adults and early childhood, Flexsoft showed significantly higher apical impedance than Standard at all time points through 60 months, whereas medial and basal segments showed no differences.

Compared with Flex28, Flexsoft generally exhibited higher impedances. Differences were significant in adults at the apical segment (1-24 months) and at medial/basal segments (6-24 months), but in early childhood only at the apical segment at 6 months.

In Flex28–Standard comparisons, adults showed higher impedance with Standard in the medial (6-24 months) and basal (6-60 months) segment, with no apical difference. In early childhood, no significant differences were detected in any segment or at any time point. Detailed demographic data are provided in Supplemental Table S3.

### Impedance Variations Between Adult and Early Childhood Participants

For apical electrodes, impedance was higher in early childhood than in adults across all electrode types and time points (Figure 4); the intraoperative between-group difference was not significant only for CI24RE(CA) and CI422. For medial electrodes, impedance was significantly higher in early childhood than in adults at all time points for CI512, Flex28 and Flexsoft. In the remaining electrode types, impedance likewise tended to be higher in early childhood, but these differences did not reach statistical significance. However, for basal electrodes, the direction of the between-group difference diverged between intraoperative and postoperative assessments. Intraoperatively, early childhood generally showed higher impedance, with significant differences for CI512, Flex28 and Flexsoft. Postoperatively, the pattern reversed, with adults tending to show higher impedance; this difference was significant at 12 months for CI422 (*P* = .0389).Detailed demographic data are provided in Supplemental Table S4.

**Figure 4.**
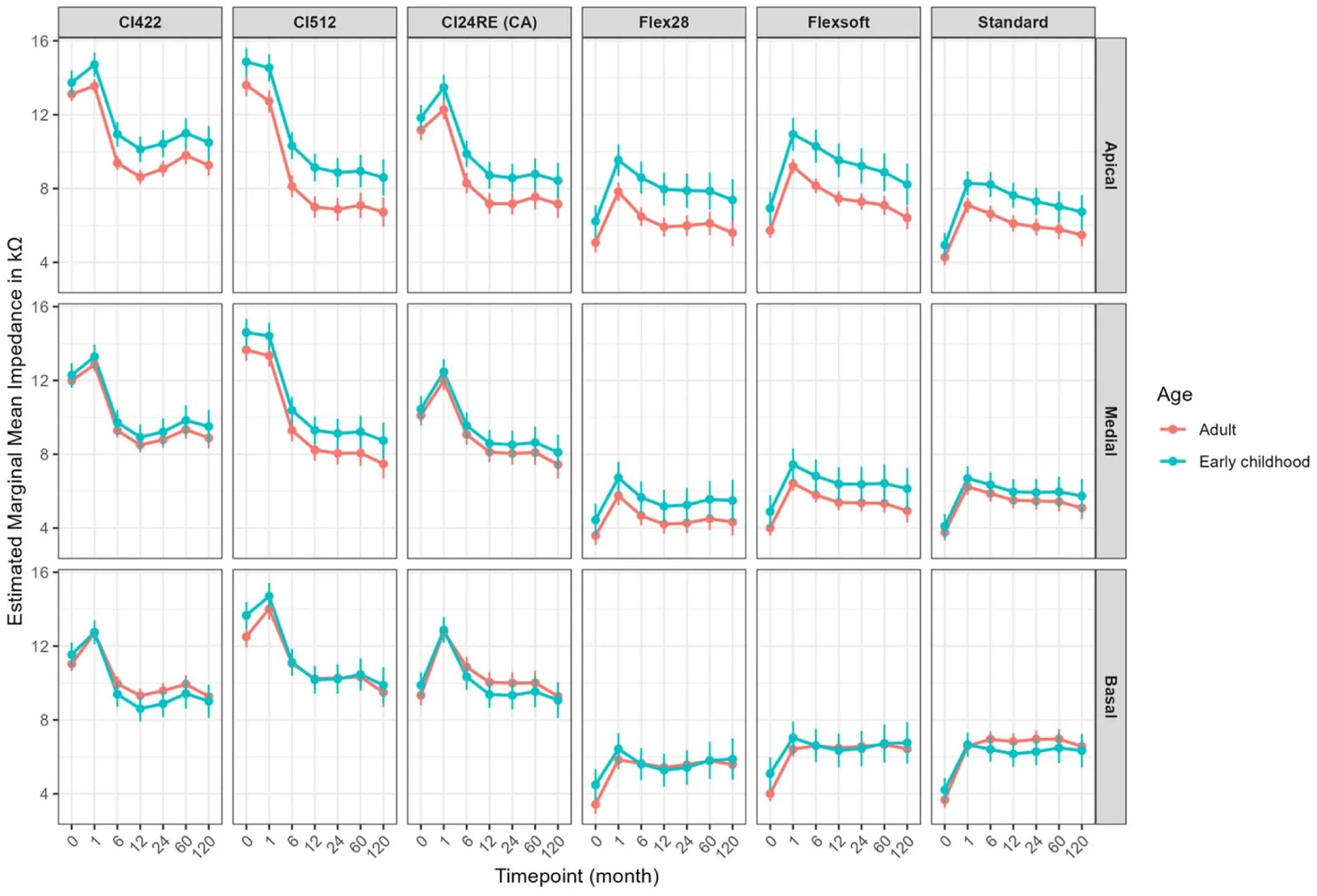
Estimated Marginal Mean impedance values (kΩ) across age groups for each electrode array type at different time points and electrode sections. Data are presented as mean ± 95% confidence interval.

## Discussion

Even minor impedance variations may warrant clinical attention because they can reflect evolving electrode–tissue interface conditions and may necessitate ongoing adjustment of device programming settings to maintain stable and comfortable stimulation. Such changes can also alter current requirements and, secondarily, affect power consumption and battery life, with potential implications for daily device use. Previous impedance studies have been limited by relatively small cohorts and restricted follow-up durations.^10,14,25,26^ Our investigation represents the first longitudinal study examining impedance patterns in a large cohort over a full 10-year period, thereby addressing a critical gap in the current literature. Notably, the differences in the trajectory of apical, medial and basal electrodes from 6 months and 120 months post-implantation underscore the persistence of long-term effects and highlight the importance of ongoing monitoring and individualized fitting over time.

### Longitudinal Impedance Dynamics

A key finding of this longitudinal study was the presence of significant impedance changes across all electrode types up to 120 months postoperatively. These results indicate that, although the acute healing phase is generally considered complete within the first 6 months post-implantation, structural tissue remodeling around the electrode array may persist over the long term.

Electric impedance seems to be primarily determined by the resistive properties of fluids and tissues surrounding the electrodes.^14,27,28^ Tissues with lower conductivity, such as fibrotic and osseous tissue, and inflammation-induced cellular responses, are associated with elevated impedance values.^11,27,29,30,31^ Impedance often increases from surgery to the initial activation, consistent with early postoperative tissue responses. Separately, the “stimulation effect” denotes a transient decrease in impedance after electrical stimulation, relative to pre-stimulation measurements.^32^ This effect has been attributed to electrochemical depassivation of platinum–iridium contacts and an increase in effective electrode surface area, thereby reducing the capacitive component of the electrode–electrolyte interface.^25,28^ Prolonged electrical stimulation may further contribute to a sustained postoperative reduction in impedance over time. An exception was observed in CI512 adults, in whom apical impedance decreased from intraoperative measurement to activation. This may reflect cohort- and device-specific factors.

Despite these insights, the mechanisms driving impedance changes during the 60 to 120 months postoperative period remain poorly understood. Long-term reductions in impedance may reflect alterations in cellular content around the electrode array, though this remains speculative.

Our findings align with and expand upon previous investigations of longitudinal impedance dynamics. Newbold et al^15^ compared impedance values at activation and again 8 to 12 years post-reporting decreased apical and increased basal electrode impedance. While the trend observed for apical electrodes is consistent with our findings, our results diverge for basal electrodes in Cochlear Nucleus arrays. This discrepancy may stem from the inclusion of deactivated electrodes in Newbold’s analysis,^15^ which were excluded in our study.

Espina González et al^33^ reported similar impedance changes in MED-EL electrodes (96.2% Standard arrays) in adult patients over a follow-up period of 5 years post-implantation, noting declines in apical and medial regions consistent with those observed in our Standard Adult group—a pattern also reported in other studies.^34^ Their slight, nonsignificant basal increase parallels our early postoperative observations, although we subsequently identified a significant decrease from 60 to 120 months. Differences in inclusion criteria, such as deactivated electrodes, may account for these discrepancies.

Brotto et al^26^ systematically examined the impedance values of CI512, CI532, and CI24RE(CA) users over 5 years and reported early stabilization after 6 months. In contrast, we observed fluctuations across cochlear regions up to 10 years post-implantation. This discrepancy may be attributed to differences in participant etiology: Brotto’s cohort consisted exclusively of patients with *GJB2* mutations causing congenital profound hearing loss, potentially contributing to the observed impedance stability.

Taken together, our findings underscore the critical importance of continued impedance monitoring beyond the early postoperative period to ensure optimal clinical outcomes. The distinct impedance changes observed in apical and basal electrodes highlight the necessity of region-specific evaluation and management during postoperative follow-up. Future studies should aim to elucidate the cellular and molecular mechanisms that shape long-term impedance trajectories in CI recipients.

### Electrode Array Type-Related Impedance Differences

The design of electrode arrays has continued to evolve with the goal of enhancing cochlear implant performance and patient outcomes. PM arrays are engineered to position contacts closer to the modiolus and spiral ganglion neurons, improving stimulation efficiency, reducing channel interaction, lowering thresholds, expanding dynamic range, and decreasing power consumption and the likelihood of non-auditory stimulation.^35,36^ In contrast, LW arrays are thinner and more flexible, reducing the risk of osseous spiral lamina fracture, spiral ganglion injury, and translocation into the scala vestibuli when properly inserted.^12,19,37^

Previous comparative studies have shown a characteristic impedance pattern: PM arrays generally exhibit higher basal impedance, while LW arrays show higher apical impedance.^10,14,19,25,38^ Our findings are broadly consistent with this framework, particularly with respect to the apical region, where LW showed higher impedance relative to PM arrays. However, the basal pattern was not uniform across PM designs and appeared phase-dependent only in the CI24RE(CA) comparison: the intraoperative direction differed from the postoperative tendency, whereas CI512 showed a stable PM-favored basal profile across time with attenuation at long-term follow-up.

These spatial-temporal trends likely reflect differences in array geometry and intracochlear positioning. The increased thickness and modiolar proximity of PM arrays may impose greater insertion-related trauma in the basal turn, potentially promoting localized fibrosis.^19^ Conversely, the more lateral position of LW arrays may provide additional intracochlear space that permits greater long-term tissue proliferation.

Within the LW group, CI422 exhibited consistently higher impedance than MED-EL arrays across regions and time points. This difference may arise from variations in materials, fabrication methods, or electrode contact surface area.

The most notable design difference between Flexsoft and Standard arrays lies in their apical configuration. Flexsoft incorporates FLEX-TIP technology with reduced apical contact surface area, producing higher impedance at these sites, consistent with our findings. Flexsoft also demonstrated higher apical impedance than Flex28 at most timepoints despite sharing the same FLEX-TIP design, likely due to Flex28’s shorter length and closer contact spacing, which may reduce electrical resistance.

Continued refinement of contact surface area, mechanical flexibility, and insertion techniques remains essential for optimizing impedance trajectories, device performance, and long-term patient outcomes.

### Adult versus Early childhood Participants

We intentionally focused on 2 implantation-age extremes that represent clinically distinct trajectories. The 7 to 17-year range is clinically heterogeneous, and many individuals in this range would transition into adulthood within a few years after implantation, which could obscure long-term impedance patterns attributable to age at implantation.

Many studies indicate that pediatric recipients tend to exhibit higher impedance values than adults.^10,19,25^ This difference may reflect physiological, anatomical, and etiological factors.

Physiologically, immature auditory pathways may reduce electrical conduction efficiency,^39^ while stronger wound-healing responses in children may promote greater fibrotic encapsulation at the electrode–tissue interface, leading to increased impedance.^19,40^

Anatomically, while the inner ear itself does not grow postnatally, subtle shifts due to skull and temporal bone growth may affect electrode positioning over time, potentially influencing long-term tissue responses and impedance trajectories in children. Besides, varying etiologies of hearing loss across age groups may influence patterns of tissue response.^19^

Interestingly, the trend of higher impedance observed in adults at the basal electrodes suggests a different underlying mechanism. One plausible explanation is that tissue growth around the electrode array originates from the cochlear entrance and extends directionally along the array, as supported by prior studies.^17,41^ However, additional factors such as age-related bone remodeling, localized calcification, or fibrosis specific to the basal turn of the cochlea may also potentially contribute to this phenomenon, although further studies are needed to confirm these mechanisms.

### Limitations

This study has several limitations. First, all implantations occurred between 2006 and 2014. Although CI technologies and clinical protocols have evolved, the electrode designs used in this cohort remain broadly comparable to contemporary devices, supporting the relevance of the physiological and postoperative processes observed. Second, despite the overall sample size, some subgroups (particularly early childhood recipients) were small, reducing statistical power for subgroup analyses. Third, because we a priori restricted the cohort to recipients implanted in early childhood (≤6 years) and adulthood (≥18 years), the findings may not generalize to individuals implanted between 7 and 17 years of age. Fourth, since our objective was to characterize long-term impedance dynamics in the typical clinical course of normally functioning implants, the cohort may underrepresent complicated postoperative courses and may underestimate real-world variability. Generalizability to recipients experiencing such events should therefore be interpreted with caution. In addition, follow-up availability varied across time points and was limited at later follow-up (particularly at 60 and 120 months), which may introduce attrition/survivor bias if missingness is non-random. Fifth, as a single-center study, the findings may reflect center-specific characteristics, including patient demographics, surgical techniques, and institutional management practices, which may limit generalizability. Finally, the number of recipients implanted with Advanced Bionics devices was insufficient for meaningful comparison across manufacturers.

## Conclusion

This decade-long longitudinal analysis reveals clear temporal patterns of electrode impedance across electrode designs and age groups, underscoring its importance for long-term cochlear implant performance. These findings indicate that electrode selection and postoperative management should incorporate factors beyond cochlear duct length to optimize outcomes. An individualized management strategy is recommended, supported by continuous impedance surveillance and carefully adjusted programming parameters. Furthermore, integrating impedance telemetry with broader clinical information—including patient history, otoscopic evaluation, and audiological assessment—may improve diagnostic accuracy and enable more precise, data-informed clinical decision-making.

## Supplemental Material

sj-csv-1-ohn-10.1177_19160216261442102 – Supplemental material for Long-Term Impedance Trends Over 10 Years Following Cochlear Implantation: A Retrospective Comparative Analysis Supplemental material, sj-csv-1-ohn-10.1177_19160216261442102 for Long-Term Impedance Trends Over 10 Years Following Cochlear Implantation: A Retrospective Comparative Analysis by Jianhang Deng, Martin Koch, Matthias Bornitz, Sijia Zhai, Marcus Neudert and Till Moritz Essinger in Journal of Otolaryngology - Head & Neck Surgery

sj-csv-2-ohn-10.1177_19160216261442102 – Supplemental material for Long-Term Impedance Trends Over 10 Years Following Cochlear Implantation: A Retrospective Comparative Analysis Supplemental material, sj-csv-2-ohn-10.1177_19160216261442102 for Long-Term Impedance Trends Over 10 Years Following Cochlear Implantation: A Retrospective Comparative Analysis by Jianhang Deng, Martin Koch, Matthias Bornitz, Sijia Zhai, Marcus Neudert and Till Moritz Essinger in Journal of Otolaryngology - Head & Neck Surgery

sj-csv-3-ohn-10.1177_19160216261442102 – Supplemental material for Long-Term Impedance Trends Over 10 Years Following Cochlear Implantation: A Retrospective Comparative Analysis Supplemental material, sj-csv-3-ohn-10.1177_19160216261442102 for Long-Term Impedance Trends Over 10 Years Following Cochlear Implantation: A Retrospective Comparative Analysis by Jianhang Deng, Martin Koch, Matthias Bornitz, Sijia Zhai, Marcus Neudert and Till Moritz Essinger in Journal of Otolaryngology - Head & Neck Surgery

sj-csv-4-ohn-10.1177_19160216261442102 – Supplemental material for Long-Term Impedance Trends Over 10 Years Following Cochlear Implantation: A Retrospective Comparative Analysis Supplemental material, sj-csv-4-ohn-10.1177_19160216261442102 for Long-Term Impedance Trends Over 10 Years Following Cochlear Implantation: A Retrospective Comparative Analysis by Jianhang Deng, Martin Koch, Matthias Bornitz, Sijia Zhai, Marcus Neudert and Till Moritz Essinger in Journal of Otolaryngology - Head & Neck Surgery

sj-csv-5-ohn-10.1177_19160216261442102 – Supplemental material for Long-Term Impedance Trends Over 10 Years Following Cochlear Implantation: A Retrospective Comparative Analysis Supplemental material, sj-csv-5-ohn-10.1177_19160216261442102 for Long-Term Impedance Trends Over 10 Years Following Cochlear Implantation: A Retrospective Comparative Analysis by Jianhang Deng, Martin Koch, Matthias Bornitz, Sijia Zhai, Marcus Neudert and Till Moritz Essinger in Journal of Otolaryngology - Head & Neck Surgery

sj-rds-1-ohn-10.1177_19160216261442102 – Supplemental material for Long-Term Impedance Trends Over 10 Years Following Cochlear Implantation: A Retrospective Comparative Analysis Supplemental material, sj-rds-1-ohn-10.1177_19160216261442102 for Long-Term Impedance Trends Over 10 Years Following Cochlear Implantation: A Retrospective Comparative Analysis by Jianhang Deng, Martin Koch, Matthias Bornitz, Sijia Zhai, Marcus Neudert and Till Moritz Essinger in Journal of Otolaryngology - Head & Neck Surgery

sj-txt-1-ohn-10.1177_19160216261442102 – Supplemental material for Long-Term Impedance Trends Over 10 Years Following Cochlear Implantation: A Retrospective Comparative Analysis Supplemental material, sj-txt-1-ohn-10.1177_19160216261442102 for Long-Term Impedance Trends Over 10 Years Following Cochlear Implantation: A Retrospective Comparative Analysis by Jianhang Deng, Martin Koch, Matthias Bornitz, Sijia Zhai, Marcus Neudert and Till Moritz Essinger in Journal of Otolaryngology - Head & Neck Surgery

sj-csv-6-ohn-10.1177_19160216261442102 – Supplemental material for Long-Term Impedance Trends Over 10 Years Following Cochlear Implantation: A Retrospective Comparative Analysis Supplemental material, sj-csv-6-ohn-10.1177_19160216261442102 for Long-Term Impedance Trends Over 10 Years Following Cochlear Implantation: A Retrospective Comparative Analysis by Jianhang Deng, Martin Koch, Matthias Bornitz, Sijia Zhai, Marcus Neudert and Till Moritz Essinger in Journal of Otolaryngology - Head & Neck Surgery

sj-txt-2-ohn-10.1177_19160216261442102 – Supplemental material for Long-Term Impedance Trends Over 10 Years Following Cochlear Implantation: A Retrospective Comparative Analysis Supplemental material, sj-txt-2-ohn-10.1177_19160216261442102 for Long-Term Impedance Trends Over 10 Years Following Cochlear Implantation: A Retrospective Comparative Analysis by Jianhang Deng, Martin Koch, Matthias Bornitz, Sijia Zhai, Marcus Neudert and Till Moritz Essinger in Journal of Otolaryngology - Head & Neck Surgery

sj-xlsx-1-ohn-10.1177_19160216261442102 – Supplemental material for Long-Term Impedance Trends Over 10 Years Following Cochlear Implantation: A Retrospective Comparative Analysis Supplemental material, sj-xlsx-1-ohn-10.1177_19160216261442102 for Long-Term Impedance Trends Over 10 Years Following Cochlear Implantation: A Retrospective Comparative Analysis by Jianhang Deng, Martin Koch, Matthias Bornitz, Sijia Zhai, Marcus Neudert and Till Moritz Essinger in Journal of Otolaryngology - Head & Neck Surgery

sj-xlsx-2-ohn-10.1177_19160216261442102 – Supplemental material for Long-Term Impedance Trends Over 10 Years Following Cochlear Implantation: A Retrospective Comparative Analysis Supplemental material, sj-xlsx-2-ohn-10.1177_19160216261442102 for Long-Term Impedance Trends Over 10 Years Following Cochlear Implantation: A Retrospective Comparative Analysis by Jianhang Deng, Martin Koch, Matthias Bornitz, Sijia Zhai, Marcus Neudert and Till Moritz Essinger in Journal of Otolaryngology - Head & Neck Surgery

sj-xlsx-3-ohn-10.1177_19160216261442102 – Supplemental material for Long-Term Impedance Trends Over 10 Years Following Cochlear Implantation: A Retrospective Comparative Analysis Supplemental material, sj-xlsx-3-ohn-10.1177_19160216261442102 for Long-Term Impedance Trends Over 10 Years Following Cochlear Implantation: A Retrospective Comparative Analysis by Jianhang Deng, Martin Koch, Matthias Bornitz, Sijia Zhai, Marcus Neudert and Till Moritz Essinger in Journal of Otolaryngology - Head & Neck Surgery

sj-xlsx-4-ohn-10.1177_19160216261442102 – Supplemental material for Long-Term Impedance Trends Over 10 Years Following Cochlear Implantation: A Retrospective Comparative Analysis Supplemental material, sj-xlsx-4-ohn-10.1177_19160216261442102 for Long-Term Impedance Trends Over 10 Years Following Cochlear Implantation: A Retrospective Comparative Analysis by Jianhang Deng, Martin Koch, Matthias Bornitz, Sijia Zhai, Marcus Neudert and Till Moritz Essinger in Journal of Otolaryngology - Head & Neck Surgery

## Data Availability

The datasets generated during and/or analyzed during the current study are available from the corresponding author on reasonable request.*

## References

[1] Dendys K Bieniasz J Bigos P Kuźnicki W Matkowski I Potyrała P. Cochlear implants—an overview. Are CIs the world’s most successful sensory prostheses? J Educ Health Sport. 2023;24(1):126-142. doi:10.12775/JEHS.2023.24.01.012

[2] Harris JM Neault MW O’Neill EE , et al. Incidence, time course, and implications of electrode abnormalities in pediatric cochlear implant recipients. Ear Hear. 2021;42(2):334-342. doi:10.1097/AUD.0000000000000924 32826503

[3] de Rijk SR Tam YC Carlyon RP Bance ML. Detection of extracochlear electrodes in cochlear implants with electric field imaging/transimpedance measurements: a human cadaver study. Ear Hear. 2020;41(5):1196-1207. doi:10.1097/AUD.0000000000000837 PMC7115972 31923041

[4] Aebischer P Meyer S Caversaccio M Wimmer W. Intraoperative impedance-based estimation of cochlear implant electrode array insertion depth. IEEE Trans Biomed Eng. 2021;68(2):545-555. doi:10.1109/TBME.2020.3006934 32746052

[5] Wimmer W Sclabas L Caversaccio M Weder S. Cochlear implant electrode impedance as potential biomarker for residual hearing. Front Neurol. 2022;13:886171. doi:10.3389/fneur.2022.886171 PMC9271767 35832176

[6] Huang CQ Tykocinski M Stathopoulos D Cowan R. Effects of steroids and lubricants on electrical impedance and tissue response following cochlear implantation. Coch Implant Int. 2007;8(3):123-147. doi:10.1179/cim.2007.8.3.123 17854099

[bibr7-19160216261442102] Wang J Sun J Sun J Chen J. Variations in electrode impedance during and after cochlear implantation: round window versus extended round window insertions. Int J Pediatr Otorhinolaryngol. 2017;102:44-48. doi:10.1016/j.ijporl.2017.08.032 29106874

[bibr8-19160216261442102] de Rijk SR Boys AJ Roberts IV , et al. Tissue-engineered cochlear fibrosis model links complex impedance to fibrosis formation for cochlear implant patients. Adv Healthcare Mater. 2023;12(24):2300732. doi:10.1002/adhm.202300732 PMC11468547 37310792

[9] Zhang L Schmidt FH Oberhoffner T , et al. Transimpedance matrix can be used to estimate electrode positions intraoperatively and to monitor their positional changes postoperatively in cochlear implant patients. Otol Neurotol. 2024;45(4):e289-e296. doi:10.1097/MAO.0000000000004145 38346796

[10] Busby P Plant K Whitford L. Electrode impedance in adults and children using the Nucleus 24 cochlear implant system. Coch Implant Int. 2002;3(2):87-103. doi:10.1179/cim.2002.3.2.87 18792117

[bibr11-19160216261442102] Tykocinski M Cohen LT Cowan RS. Measurement and analysis of access resistance and polarization impedance in cochlear implant recipients. Otol Neurotol. 2005;26(5):948-956. doi:10.1097/01.mao.0000185056.99888.f3 16151342

[bibr12-19160216261442102] Wilk M Hessler R Mugridge K , et al. Impedance changes and fibrous tissue growth after cochlear implantation are correlated and can be reduced using a dexamethasone eluting electrode. Yamamoto M, ed. Plos One. 2016;11(2):e0147552. doi:10.1371/journal.pone.0147552 PMC4739581 26840740

[bibr13-19160216261442102] Choi J Payne MR Campbell LJ , et al. Electrode impedance fluctuations as a biomarker for inner ear pathology after cochlear implantation. Otol Neurotol. 2017;38(10):1433-1439. doi:10.1097/MAO.0000000000001589 29135865

[14] Leone CA Mosca F Grassia R. Temporal changes in impedance of implanted adults for various cochlear segments. Acta Otorhinolaryngol Ital. 2017;37(4):312-319. doi:10.14639/0392-100X-1471 PMC5584104 28872161

[15] Newbold C Risi F Hollow R Yusof Y Dowell R. Long-term electrode impedance changes and failure prevalence in cochlear implants. Int J Audiol. 2015;54(7):453-460. doi:10.3109/14992027.2014.1001076 25766491

[16] Konrad S Framke T Kludt E Büchner A Lenarz T Paasche G. Do impedance changes correlate with a delayed hearing loss after Hybrid L24 implantation? Ear Hear. 2021;42(1):163-172. doi:10.1097/AUD.0000000000000914 32769433

[17] Konrad S Büchner A Lenarz T Paasche G. Impedance development after implantation of hybrid-L24 cochlear implant electrodes. Int J Audiol. 2023;62(12):1137-1144. doi:10.1080/14992027.2022.2125914 36193989

[18] Hu H Chen JK Li LP , et al. Evolution of impedance values in two different electrode array designs following activation of cochlear implants 1 day after surgery: a study of 58 patients. Clin Otolaryngol. 2020;45(4):584-590. doi:10.1111/coa.13327 30864257

[bibr19-19160216261442102] Velandia S Martinez D Goncalves S , et al. Effect of age, electrode array, and time on cochlear implant impedances. Coch Implant Int. 2020;21(6):344-352. doi:10.1080/14670100.2020.1788859 32640889

[20] Pauna HF Do Amaral MSA Fonseca DS , et al. The influence of cochlear volume on temporal changes of impedance among cochlear implant patients. OHBM. 2023;4(1):3. doi:10.3390/ohbm4010003

[21] Bates D Mächler M Bolker B Walker S. Fitting linear mixed-effects models using lme4. J Stat Soft. 2015;67(1):1-48. doi:10.18637/jss.v067.i01

[22] Akaike H. A new look at the statistical model identification. IEEE Trans Automat Contr. 1974;19(6):716-723. doi:10.1109/TAC.1974.1100705

[23] Nakagawa S Schielzeth H. A general and simple method for obtaining *R*^2^ from generalized linear mixed-effects models. O’Hara RB , ed. Methods Ecol Evol. 2013;4(2):133-142. doi:10.1111/j.2041-210x.2012.00261.x

[24] Lenth RV. Least-squares means: the *R* package lsmeans. J Stat Soft. 2016;69(1):1-33. doi:10.18637/jss.v069.i01

[25] Molisz A Zarowski A Vermeiren A , et al. Postimplantation changes of electrophysiological parameters in patients with cochlear implants. Audiol Neurotol. 2015;20(4):222-228. doi:10.1159/000377615 25969138

[26] Brotto D Caserta E Sorrentino F , et al. Long-term impedance trend in cochlear implant users with genetically determined congenital profound hearing loss. J Am Acad Audiol. 2022;33(2):105-114. doi:10.1055/s-0041-1739290 35577055

[27] Newbold C Richardson R Huang CQ Milojevic D Cowan R Shepherd R. An in vitro model for investigating impedance changes with cell growth and electrical stimulation: implications for cochlear implants. J Neural Eng. 2004;1(4):218-227. doi:10.1088/1741-2560/1/4/005 15876642

[28] Newbold C Richardson R Millard R Seligman P Cowan R Shepherd R. Electrical stimulation causes rapid changes in electrode impedance of cell-covered electrodes. J Neural Eng. 2011;8(3):036029. doi:10.1088/1741-2560/8/3/036029 PMC3147028 21572219

[29] Clark GM Shute SA Shepherd RK Carter TD. Cochlear implantation: osteoneogenesis, electrode-tissue impedance, and residual hearing. Ann Otol Rhinol Laryngol Suppl. 1995;166:40-42. 7668722

[30] Duan YY Clark GM Cowan RSC . A study of intra-cochlear electrodes and tissue interface by electrochemical impedance methods in vivo. Biomaterials. 2004;25(17):3813-3828. doi:10.1016/j.biomaterials.2003.09.107 15020157

[31] Ishai R Herrmann BS Nadol JB Quesnel AM. The pattern and degree of capsular fibrous sheaths surrounding cochlear electrode arrays. Hear Res. 2017;348:44-53. doi:10.1016/j.heares.2017.02.012 PMC5738657 28216124

[32] Paasche G Tasche C Stöver T Lesinski-Schiedat A Lenarz T. The long-term effects of modified electrode surfaces and intracochlear corticosteroids on postoperative impedances in cochlear implant patients. Otol Neurotol. 2009;30(5):592-598. doi:10.1097/MAO.0b013e3181ab8fba 19546829

[33] Espina González C Morant Ventura A Pla Gil I Aragonés Redó M Pérez Carbonell T Marco Algarra J . Variation of electrical impedance over 5 years post-implantation and relationship with the maximum comfort level (MCL) in adults with cochlear implants. Acta Otorrinolaringol (English Edition). 2024;75(1):23-30. doi:10.1016/j.otoeng.2024.01.004 38224870

[34] Henkin Y Kaplan-Neeman R Kronenberg J Migirov L Hildesheimer M Muchnik C. Electrical stimulation levels and electrode impedance values in children using the MED-EL Combi 40+ cochlear implant: a one year follow-up. J Basic Clin Physiol Pharmacol. 2005;16(2-3):127-138. doi:10.1515/JBCPP.2005.16.2-3.127 16285465

[35] Shepherd RK Hatsushika S Clark GM. Electrical stimulation of the auditory nerve: the effect of electrode position on neural excitation. Hear Res. 1993;66(1):108-120. doi:10.1016/0378-5955(93)90265-3 8473242

[36] Lee JY Hong SH Moon IJ , et al. Effect of cochlear implant electrode array design on electrophysiological and psychophysical measures: lateral wall versus perimodiolar types. J Audiol Otol. 2019;23(3):145-152. doi:10.7874/jao.2019.00164 PMC6646897 31315391

[37] Bas E Bohorquez J Goncalves S , et al. Electrode array-eluted dexamethasone protects against electrode insertion trauma induced hearing and hair cell losses, damage to neural elements, increases in impedance and fibrosis: a dose response study. Hear Res. 2016;337:12-24. doi:10.1016/j.heares.2016.02.003 26892906

[38] Sanderson AP Rogers ETF Verschuur CA Newman TA. Exploiting routine clinical measures to inform strategies for better hearing performance in cochlear implant users. Front Neurosci. 2019;12:1048. doi:10.3389/fnins.2018.01048 PMC6340939 30697145

[39] Brkic FF Umihanic S Harcinovic A Piric L Brkic F. High electrode impedance values in pediatric cochlear implant recipients may imply insufficient auditory and language skills development. JCM. 2020;9(2):506. doi:10.3390/jcm9020506 PMC7074351 32069787

[40] Lago JC Puzzi MB. The effect of aging in primary human dermal fibroblasts. Picardo M, ed. Plos One. 2019;14(7):e0219165. doi:10.1371/journal.pone.0219165 PMC6608952 31269075

[41] Paasche G Bockel F Lesinski-Schiedat A Lenarz T. Changes of postoperative impedances in cochlear implant patients: the short-term effects of modified electrode surfaces and intracochlear corticosteroids. Otol Neurotol. 2006;27(5):639-647. doi:10.1097/01.mao.0000227662.88840.61 16868511

